# Altered SIgA-targeting of gut microbiota is associated with long-term dysbiosis in pediatric solid organ transplant recipients

**DOI:** 10.1080/19490976.2026.2675078

**Published:** 2026-05-24

**Authors:** Kora Schulze, Imeke Goldschmidt, Anette Melk, Martin Boehne, Sabrina Woltemate, Matthias Ballmaier, Svea Kleiner, Elena Lehmann, Miriam Kramer, Marius Vital

**Affiliations:** a Institute for Medical Microbiology and Hospital Epidemiology, Hannover Medical School, Hannover, Germany; b Department of Pediatric Liver, Kidney and Metabolic Diseases, Division of Pediatric Hepatology and Liver Transplantation, Hannover Medical School, Hannover, Germany; c Department of Pediatric Liver, Kidney and Metabolic Diseases, Hannover Medical School, Hannover, Germany; d Department of Pediatric Cardiology and Intensive Care Medicine, Hannover Medical School, Hannover, Germany; e Central Research Facility Cell Sorting, Hannover Medical School, Hannover, Germany; f KlinStrucMed Program, Dean's Office for Academic Career Development, Hannover Medical School, Hannover, Germany; g German Center for Infection Research (DZIF), Hannover-Braunschweig, Germany; h Cluster of Excellence RESIST (EXC 2155), Hannover Medical School, Hannover, Germany

**Keywords:** Microbiota, IgA, solid organ transplantation, immunosuppression, metagenomics

## Abstract

The composition of the gut microbiota (GM) is altered in solid organ transplantation (SOT) recipients, where the degree of dysbiosis is associated with long-term survival and is believed to be influenced by immunosuppression therapy. At the interface stands secretory (S)IgA, however, little is known about its role in governing dysbiosis in the context of SOT. We performed quantitative metagenomic analyses of the GM accompanied by SIgA sequencing in 48 pediatric SOT recipients (age = 10.6 ± 4.7 y) receiving either heart (*n* = 11), kidney (*n* = 10) or liver transplantation (*n* = 27), and compared the results to age-matched healthy controls (HC, *n* = 16). We confirmed compositional and functional dysbiosis in SOT recipients, with the degree of dysbiosis being associated with tacrolimus (TAC) levels. Overall, SOT recipients exhibited higher SIgA levels than HC, along with an increased percentage of bacteria targeted and altered target spectra. Furthermore, altered SIgA responses were associated with the degree of dysbiosis. A mechanistic model connecting immunosuppression, GM composition and SIgA-targeting is proposed, suggesting that GM dysbiosis in SOT recipients is mediated by the immune system through the SIgA response; direct drug-mediated effects on fecal communities were not observed in *in vitro* experiments. Our study provides new insights into factors that contribute to persisting dysbiosis in SOT recipients.

## Graphical abstract

**Figure:**
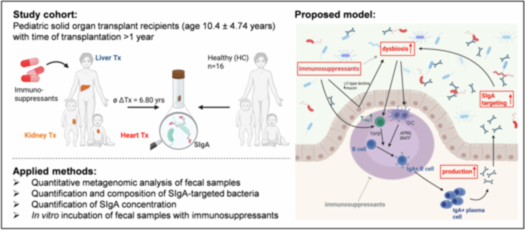

## Introduction

Solid organ transplantation (SOT) is the final therapeutic option in patients suffering from severe diseases with irreversible and life-endangering progression that cannot be treated otherwise.^1^ Subsequent immunosuppression is required throughout life to prevent graft rejection. The dose and regimen of immunosuppressants are adapted based on the organ transplanted, the time since transplantation, the age of the patients as well as individual factors and require a tight therapeutic monitoring after surgery. While strong and often repeated antibiotic treatments before and short-term after SOT are known to cause gut microbiota (GM) dysbiosis,^2^ there is increasing evidence that also immunosuppressants decrease the bacterial richness and diversity, potentially causing altered GM composition also several years post-transplantation.^2^ ^-^ ^4^ Moreover, studies showed that GM are capable of modulating drug pharmacokinetics, for example, by altering transporter expression or metabolizing the drug itself, highlighting the bidirectional relationship between immunosuppression and GM.^5^ ^,^ ^6^ Evidence related to the effects of SOT and immunosuppression on GM is scarce and very heterogeneous with regard to the study design and the characteristics of the analyzed patient cohort. Furthermore, the extrapolation of results from adults to children is limited, as pediatric SOT recipients usually have different underlying diseases as indications for transplantation, often require a more dynamic immunosuppressant regimen and show a higher risk and frequency of infection and transplant-related complications.^7^ ^-^ ^10^ As observed by us and others, GM dysbiosis in pediatric SOT recipients is associated with short- and long-term health outcomes, including an increased risk for infection, graft health and all-cause mortality, highlighting the importance to combat GM dysbiosis in those patients.^2^ ^,^ ^11^ ^,^ ^12^

The interaction between the host immune system and the GM is regulated to a large extent via secretory immunoglobulin A (SIgA). SIgA is the most abundant immunoglobulin isotype in mammals and is predominantly located at mucosal surfaces, where its main function is the exclusion of pathogens.^13^ ^,^ ^14^ However, recent years revealed that it is also targeting commensal bacteria, thereby regulating their colonization, growth, and motility.^15^ ^,^ ^16^ The underlying mechanisms by which SIgA exerts its various effects on GM are hypothesized to be the result of SIgA affinity maturation and consequent differences in binding to the respective bacteria, as outlined in several reviews on this topic.^17^ ^,^ ^18^ SIgA-targeting of GM and its impact on host immunity are also of great importance for child development. During the first days of life, maternal breastmilk is the only source of SIgA for the neonate, eliciting an important role in the early shaping of infants' GM.^19^ ^,^ ^20^ Ding et al.^21^ reviewed SIgA-targeting patterns and consequent changes in microbiota composition during child development, as well as factors influencing this relation. The authors highlighted the role of SIgA in the maturation of the infants' immune system and connected altered SIgA-targeting to diseases later in life, such as allergies, diarrhea and colitis. The association between altered SIgA-targeting and disease has also been intensively investigated in adults, where it has often been associated with infection and inflammation along with overall GM dysbiosis.^16^ ^,^ ^22^

Immunosuppression in SOT recipients targets B and T cells, which are also involved in the synthesis and maturation of SIgA. However, whether those patients show an altered SIgA expression as well as subsequent altered SIgA-targeting of GM that eventually contributes to the observed dysbiosis, is unknown. To test this hypothesis, we investigated the SIgA-targeting of GM in pediatric SOT recipients under stable immunosuppression and compared the results to an age-matched healthy cohort, providing the first study to investigate the effect of immunosuppression on SIgA-targeting of GM and its relation to dysbiosis in patients after SOT. We compared different organ transplants (heart, liver, and kidney) as well as different immunosuppressant regimens from a major German transplantation center, thereby minimizing regional variability in the GM and differences in surgical and post-surgery management.

## Methods

### Study cohort and sample collection

All patients were recruited at the pediatric hospital at Hannover Medical School. Inclusion criteria were the transplantation of a solid organ (heart, kidney or liver) with the time of transplantation >1 y. The exclusion criteria were antibiotic therapy within one month before sampling and any diseases affecting gastrointestinal health, bile acid metabolism or pancreatic secretion. Control samples were selected based on age from an existing collection^2^ to achieve an age-matched control group. Fecal samples were collected and divided into a naïve aliquot and an aliquot stored in a DNA/RNA shield (ZYMO, USA) by the participants either at home or directly during a hospital visit and stored continuously at −20 °C until processing.

### Processing of fecal samples for flow cytometric analyses

The bacterial number (cells per gram stool) was measured as described in Kircher et al.^23^ Subsequently three aliquots were taken for SIgA processing, one aliquot served for flow cytometric quantification of SIgA-targeted bacteria, one aliquot was used for cell sorting, and the third aliquot served as an (unlabeled) control. Pre-experiments revealed that the ratio between the number of cells and the antibody concentration is a crucial factor for obtaining reliable results, and each aliquot thus contained 50 million bacteria as this was identified as the optimal number (Supplementary Figure S1). All aliquots were centrifuged at 8000 × *g* at 4 °C for 5 min, and the supernatant was discarded. The samples were washed with cold staining buffer (1 x PBS, 1% BSA, 2 mM EDTA) and the supernatant was again discarded. The bacterial pellet was blocked for 20 min on ice using mouse serum (1:10) before being incubated with APC-conjugated anti-human IgA (1:10, Miltenyi, Germany) in the case of the first two aliquots and with 1 x PBS (1:10) in the case of the control aliquot for 30 min covered on ice. Following incubation, the cells were washed twice with cold staining buffer, and the supernatant was discarded. The bacterial pellets were resuspended and incubated with SYBR Green I (Thermo Fisher Scientific, USA) and 0.5 mM EDTA for 15 min at RT in the dark. The aliquot for sorting was stored covered on ice and sorted within one hour, whereas the aliquot for quantification was immediately measured at a Northern Lights flow cytometer (Cytek, USA). The percentage of SIgA coated bacteria was quantified by plotting the signals detected from SYBR Green I emission (all bacteria) against the signal detected from the APC-conjugated anti-IgA (SIgA coated bacteria). The measurements from the control sample served to set the gate accordingly. An example of the gating is shown in Supplementary Figure S2.

### Flow cytometric cell sorting of bacteria

Bacterial populations were flow cytometrically sorted into IgA-positive and IgA-negative fractions, based on staining with an anti-IgA antibody as described above. A total of 500,000 cells were sorted for each fraction using a FACSAria Ilu cell sorter (Becton-Dickinson, USA) with a 70 µm nozzle. After sorting, both fractions were reanalyzed and resorted if the purity within a fraction was below 80%.

### Sequencing and bioinformatics analyses

DNA was extracted using the ZymoBIOMICS Miniprep Kit (ZYMO, USA) for the naïve samples and the ZymoBIOMICS Mikroprep Kit (ZYMO, USA) for the sorted fractions according to the manufacturer's protocols. Library preparation for 16S rRNA gene sequencing (sorted fractions and naïve samples) and metagenomics shotgun sequencing (only naïve samples) and subsequent sequencing on MiSeq and NovaSeq6000, respectively, were done as described by Kircher et al.^23^ Bioinformatic analysis of amplicons (16S rRNA gene) was performed as described in reference ^23^, whereas analyses of shotgun data was done according to reference ^24^ yielding compositional and functional data.

### Measurement of SIgA fecal concentration

The total fecal SIgA concentration and the proportion of bound and unbound SIgA were quantified using both the whole filtrate and the supernatant of 1:10 diluted naïve fecal samples in PBS based on an IgA uncoated human Enzyme-Linked Immunosorbent Assay (ELISA, Thermo Fisher Scientific, USA) according to the manufacturer's instruction. Concentration in the filtrate was indicating total fecal SIgA, whereas the concentration in the supernatant was attributed to unbound SIgA. The concentration of bound SIgA was calculated by subtracting the unbound SIgA values from those of the total SIgA.

### *In vitro* incubation of fresh fecal samples with immunosuppressants

To investigate whether immunosuppressants have a direct effect on GM composition, *in vitro* cultivation of fecal communities from fresh stool samples was done according to the protocol described by Arnold et al.^25^ using 2 g L^−1^ glucose as the substrate and drugs at the following concentrations: 1 mg L^−1^ TAC, 10 mg L^−1^ TAC, 10 mg L^−1^ CSA, and 100 mg L^−1^ CSA. Concentrations were chosen to mimic the *in vivo* situation, reflecting the highest dose administered and taking into account that TAC is administered in a lower dose compared with CSA. As immunosuppressants are rapidly absorbed along the entire GI system, but predominantly in the upper intestine,^26^ ^,^ ^27^ a second, reduced concentration was included to more closely mimic the actual portion reaching the colon. At the end of the 24 h growth period, aliquots were taken for flow cytometric quantification of the total bacterial cell number and 16S rRNA gene sequencing using the protocols described by Arnold et al.^25^ The results were compared to those of replicates grown in plain media (“naive”) as well as to those of replicates grown with 1% DMSO in order to discriminate the effects of immunosuppressants from the effects of the solvent.

### Statistical analysis

Hierarchical clustering was done by k-means analyses based on Bray–Curtis dissimilarities (BC) at the species level using R statistical software (version 4.3.3, R foundation for statistical Computing, Vienna, Austria) with the *cluster* (v2.1.6) and *purrr* package (v1.0.2). Metric dimensional scaling analysis was done on BC dissimilarities at the species level for the metagenomics data and at the genus level for the 16S rRNA gene sequencing data using the *phyloseq* package (v1.44.0). Pairwise PERMANOVA analyses were performed with *pairwise Adonis* (v0.4.1) and the *vegan* package (v2.6-6.1) on BC dissimilarities (genus level). GM diversity was compared between groups by calculating the Shannon index, the number of observed species, and the BC distance to the mean BC dissimilarity in HC (BC to HC) for each sample. For differential abundance calculations of taxa, the relative abundance of a taxon was compared to the mean relative abundance of the respective taxon within HC using linear regression analyses (function *lm*; log(data + 1)) and corrected for multiple testing using a local false discovery rate (*lfdr*) < 0.05, calculated via the *fdrtool* package (v1.2.18). A *lfdr* < 0.1 was considered as a trend. The general characteristics of patients, diversity and SIgA parameters were compared between groups using the *lm* function of the package fdrtool (v1.2.18) feeding log-transformed data (log(data + 1)) and controlling for the time since transplantation. For all the parameters, a *p*-value < 0.05 was considered significant, whereas a *p*-value < 0.1 was reported as a trend. On the immunosuppressant level, organ was included as a random effect for comparison between parameters using the *lmer* function of the package lme4 (v1.1.36). The selected parameters were correlated in R using regression analyses (function *lm*) feeding log-transformed data (log(data + 1) for continuous variables. The SIgA binding probability (SIgA-bP) was calculated according to the equation given in reference.^28^ BC to HC based on SIgA-bP was calculated as done on compositional level considering the SIgA-bP of taxa with a prevalence of >80% across all samples. For analyses of individual taxa, significance was based on *lfdr*, and differences with an original *p-*value < 0.01 (and a *lfdr* > 0.05) are reported as well. Boxplots and scatter plots were created with GraphPad Prism (version 8.4.3), and Figure 6 was created with BioRender.com. For the analysis of the *in vitro* results, samples incubated with immunosuppressants were compared with a 1% DMSO control sample to discriminate between the effect of the immunosuppressant and the effect of the solvent using PERMANOVA analyses, with the donor included as a random effect. For differential abundance calculations of taxa, the relative abundance of a taxon was compared to the mean relative abundance of the respective taxon within the 1% DMSO control using linear regression analyses as described above. Bacterial cell counts, the Shannon index and the number of observed species were compared between groups using the *lmer* function as described above, and including the donor as a random effect.

## Results

### Description of the study cohort

Stool samples from 48 patients (age = 10.4 ± 4.74 y) and 16 age-matched healthy controls (HC; age = 10.2 ± 4.81 y) were analyzed. All patients had undergone solid organ transplantation (SOT), with *n* = 11 receiving a heart, *n* = 10 receiving a kidney and *n* = 27 receiving a liver transplant. Samples were collected on average 6.80 ± 4.19 y after transplantation. A detailed overview of patient characteristics and medication is provided in Table 1. The majority of patients (*n* = 34) received tacrolimus (TAC) as the primary immunosuppressant, whereas the other 14 patients received cyclosporine A (CSA). Liver transplant recipients (LTR) were treated with either TAC or CSA as monotherapy, whereas kidney (KTR) and heart transplant recipients (HTR) were also on steroids; most of the latter patients (*n* = 15) additionally received a purine-analogue or an mTOR inhibitor as a third immunosuppressant.

**Table 1.** Characteristics of the study population.

		Patients (*n* = 48)	Healthy (*n* = 16)
Sex (female, *n*)		23	5
Age (years)		10.6 ± 4.7	10.2 ± 4.8
Organ transplanted (*n*)			
	Liver	27	
	Heart	11	
	Kidney	10	
Time since transplantation (years)		6.8 ± 4.2	
Immunosuppressant			
a. Total number			
	*n* = 1	27	
	*n* = 2	6	
	*n* = 3	15	
b. Type (*n*)			
	Tacrolimus (TAC)	34	
	Cyclosporin A (CSA)	14	
	Everolimus	9	
	Sirolimus	1	
	Mycophenolate Mofetil (MMF)	4	
	Azathioprin	1	
	Prednisolon	21	
c. Level [µl/l]			
	TAC	4.94 ± 2.55	
	CSA	46.4 ± 15.9	
	Everolimus	3.97 ± 1.09	
	Sirolimus	10.1	
d. Dose [mg]			
	MMF	685 [100–1000]	
	Azathioprin	10	
	Prednisolon	3.18 [1−5]	

### Patients clustered into two groups based on GM composition

**Figure 1.**
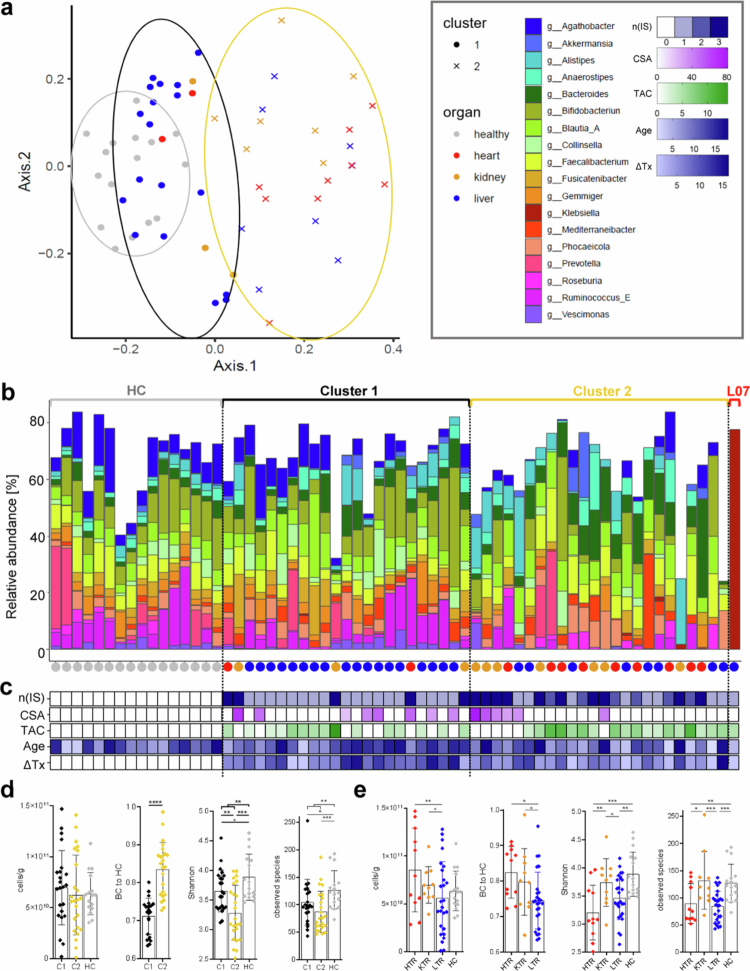
Comparison of bacterial communities between solid organ transplantation (SOT) patients and healthy controls (HC). Panel a depicts the metric multidimensional scaling analysis of all samples based on Bray–Curtis dissimilarities (BC) of metagenomics sequencing data on the species level. Clustering was done by the k-means algorithm, with colored circles indicating the respective clusters (black = C1 (*n* = 23), gold = C2 (*n* = 24), gray = HC (*n* = 16)). The samples are additionally colored according to the respective organ group. Panel b shows the relative abundance of major genera (mean relative abundance > 1%) for each sample grouped by the identified clusters shown in panel a. The colored dots below the x-axis indicate the respective organ group of each sample. The sample L07 was allocated outside the identified two clusters and is shown separately. In panel c the information on the number of administered immunosuppressants (n(IS)), blood levels of tacrolimus (TAC) and cyclosporine A (CSA), age and time since transplantation (ΔTx) for each sample is given; white color indicates no data. The distribution of bacterial cell counts, the BC distance of each sample to the mean BC in HC (representing the degree of dysbiosis; BC to HC), the Shannon index and the number of observed species per cluster and organ group are given in panels d and e, respectively. HTR = heart transplant recipients, KTR = kidney transplant recipients, LTR = liver transplant recipients.^+^ *p* < 0.1, *p < 0.05, **p < 0.01, ***p < 0.001, ****p < 0.0001.

Metagenomic analysis based on the species level grouped samples into two distinct clusters (C1 and C2) as determined by k-means clustering analysis based on Bray–Curtis dissimilarities (BC) (Figure 1a, Supplementary Figure S3). Cluster 1 included all healthy samples and the majority of samples from LTR (Figure 1a, dots). For all the following analyses, however, C1 refers only to patients within that cluster (*n* = 23), comprising *n* = 18 LTR, *n* = 3 KTR, and *n* = 2 HTR, whereas samples derived from healthy children were treated as a separate (control) group (HC, *n* = 16) (Figure 1b). The samples in C2 (*n* = 24) comprised most KTR (*n* = 7) and HTR (*n* = 9) as well as *n* = 8 LTR. C2 displayed a higher dysbiosis based on BC distance to the mean BC of HC (0.835 ± 0.067, BC to HC) compared with samples from C1 (0.711 ± 0.048, *p < *0.0001) (Figure 1a and d). There was no difference in the age distribution between any cluster and HC (Figure 1c); however, patients in C2 had a non-significant trend towards a lower time since transplantation (ΔTx, 5.82 ± 3.91 y) compared with C1 (8.08 ± 4.14 y, *p = *0.0918). Consequently, time since transplantation was included as a fixed effect variable in all further analyses. No differences in TAC or CSA levels were observed between the two patient clusters. When comparing organs, TAC levels were higher in both HTR (6.69 ± 2.38 µl/l, *p = *0.0027) and KTR (6.32 ± 4.33 µl/l, *p = 0.0414*) compared with LTR (3.77 ± 1.32 µl/l). CSA levels did not differ between organ groups. One LTR sample (“L07”) was located far outside the two identified clusters displaying a strong dominance of the genus Klebsiella (77.7%) (Figure 1b); this sample was, hence, excluded from all further statistical analyses.

The total bacterial cell count was similar between the two clusters but was higher in HTR (8.57 × 10^10^ ± 4.37 × 10^10^ cells/g) compared with LTR (5.62 × 10^10^ ± 3.79 × 10^10^ cells/g, *p = *0.0091) (Figure 1d and e). The bacterial cell counts were not different in any organ group when compared with HC. HTR had a larger BC to HC (0.824 ± 0.072) compared with LTR (0.745 ± 0.079, *p = *0.0148) (Figure 1e). KTR also showed a slightly elevated BC to HC (0.797 ± 0.094) compared with LTR, however, this observation only trended to be statistically significant (*p = *0.0877). C2 was characterized by a reduced Shannon index (3.28 ± 0.46) compared with those of HC (3.89 ± 0.39, *p = *0.0001) and C1 (3.65 ± 0.35, *p = *0.0050) (Figure 1d). Moreover, both clusters had a reduced number of observed species (C1: 105 ± 42, *p = *0.0436; C2: 87.3 ± 37.4, *p = *0.0007) compared with HC (127 ± 34.8) (Figure 1d). On the organ level, HTR had a reduced diversity compared with HC as indicated by a lower Shannon index (HTR: 3.21 ± 0.49; HC: 3.89 ± 0.39, *p = *0.0004) as well as a lower number of observed species (HTR: 89.9 ± 36.2; HC: 127 ± 34.8, *p = *0.0052). Similar results were found in LTR (Shannon: 3.46 ± 0.39, *p = *0.0017; observed species: 84.5 ± 27.9, *p = *0.0001). KTR did not show differences in diversity compared with HC but had higher a Shannon index (3.46 ± 0.39, *p = *0.0061) and a higher number of observed species (132 ± 52.9, *p = *0.0230) compared with HTR, the latter was also being higher compared with LTR (*p = *0.0008) (Figure 1e). A higher Shannon index in KTR compared with LTR was observed as a non-significant trend (*p = *0.0620).

**Figure 2.**
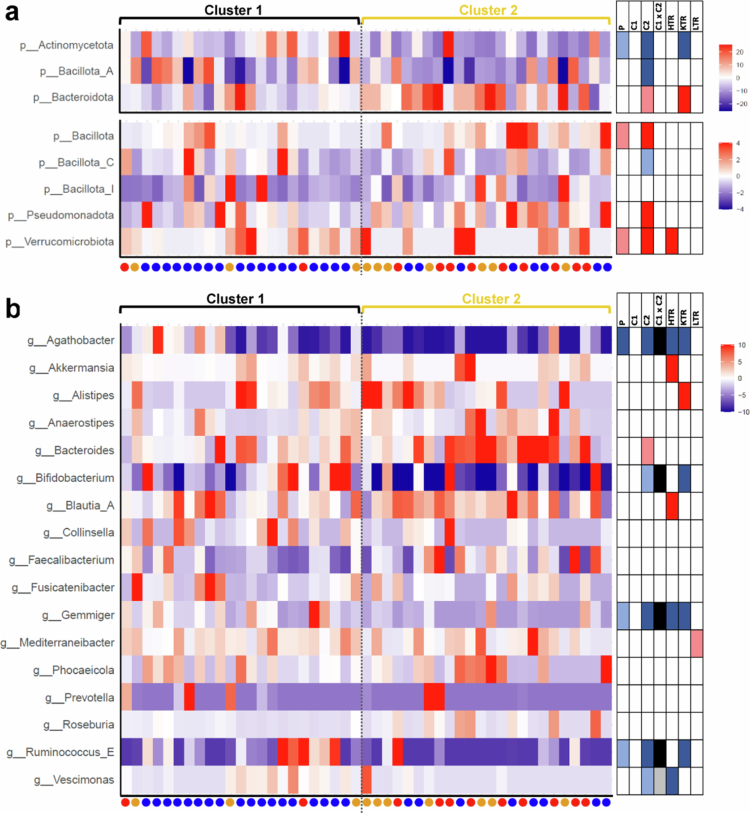
Differences in the relative abundance of taxa in patient samples compared with healthy controls (HC, mean relative abundance). Taxa with a mean relative abundance > 1% are shown on the phylum level (panel a) and the genus level (panel b). Samples are ordered according to Figure 1b. Dots below the x-axis indicate the respective organ group of each sample, color coding is according to Figure 1. The tables on the right give information about differences for the respective group with red and blue colors indicating a higher and lower relative abundance, respectively, compared with HC. Dark colors indicate significant differences (*lfdr* < 0.05), whereas light colors indicate non-significant trends (*lfdr* < 0.1). For comparison between clusters (C1 × C2), black and gray color indicate significant and trending lower abundance, respectively, of a taxon in C2 compared with C1. HTR = heart transplant recipients, KTR = kidney transplant recipients, LTR = liver transplant recipients.

To further analyze specific differences in GM composition, we compared the relative abundance of individual taxa between groups. GM composition on the phylum level did not differ in patients compared with HC (Figure 2a). Analyses of major genera (mean relative abundance > 1%) showed a reduced relative abundance of the taxon *Agathobacter* (*lfdr = *0.0080) in patients compared with HC (Figure 2b). The dominant species of this genus, *Agathobacter rectalis,* was also lower abundant in patients (*lfdr = *0.0348). Additionally, there was a non-significant trend towards lower abundances of *Gemmiger* (*lfdr = *0.0602) and *Ruminococcus_E* (*lfdr = *0.0991) in patients compared with HC (Supplementary Table S1). When comparing clusters, C2 was characterized by lower relative abundances of bacteria belonging to the phyla *Actinomycetota* and *Bacillota_A* and higher abundances of bacteria belonging to the phyla *Bacillota*, *Pseudomonadota,* and *Verrucomicrobiota* compared with HC (all: *lfdr = *0.0236). Furthermore, C2 was characterized by reduced abundances of the genera *Agathobacter* (with HC: *lfdr = *0.0001, with C1: *lfdr = *0.0109), *G* *emmiger* (with HC: *lfdr = *0.0001, with C1: *lfdr = *0.0096) and *Ruminococcus_E* (with HC: *lfdr = *0.0087, with C1: *lfdr = *0.0195) compared with both HC and C1. The dominant species of those genera (relative abundance of >0.1%) were *Agathobacter rectalis, Gemmiger qucibialis,* and *Ruminococcus_E bromii*, respectively, with the first two also being lower abundant in C2 compared with HC (*A.r.: lfdr = *0.0003, *G.q.: lfdr = *0.0041) and C1 (*A.r.: lfdr = *0.0.0256, *G.q.: lfdr = *0.0.0256). Furthermore, C2 trended towards lower relative abundances of *Bifidobacterium* and *Vescimonas*, and a higher relative abundance of *Bacteroides* (all: *lfdr = *0.0503) (Figure 2b). Regarding the former, several highly abundant species, namely, *B. adolescentis* (*lfdr < *0.0001), *B. catenulatum* (*lfdr = *0.0333), and *B. infantis* (*lfdr = *0.0041), were reduced in C2 compared with HC (Supplementary Table S1). None of the major genera had a different relative abundance in C1 compared with HC (Figure 2b).

Analyses on the organ level showed higher abundances of the phylum *Verrucomicrobiota* (*lfdr = *0.0276) and the genera *Akkermansia* (*lfdr = *0.0432) and *Blautia_A* (*lfdr = *0.0016) as well as lower abundances of *Agathobacter* (*lfdr = *0.0090), *Gemmiger* (*lfdr = *0.0016), and *Vescimonas* (*lfdr = *0.0432) in HTR compared with HC (Figure 2b). KTR showed a lower relative abundance of *Actinomycetota* (*lfdr = *0.0079) and a higher relative abundance of *Bacteroidota* (*lfdr = *0.0293) compared with HC. Bacteria belonging to the genus *Alistipes* were more abundant in KTR compared with HC (*lfdr = *0.0175), whereas *Agathobacter* (*lfdr = *0.0063), *Bifidobacterium* (*lfdr = *0.0157), *Gemmiger* (*lfdr = *0.0370), and *Ruminococcus_E* (*lfdr = *0.0254) were lower abundant in this group. *Mediterraneibacter* trended towards higher abundance in LTR (*lfdr = *0.0981), and none of the other analyzed taxa was differently abundant in this group compared with HC. Overall, HTR had the largest number of differently abundant taxa of all organ groups compared with HC. GM of LTR resembled most closely the composition found in HC with the lowest number of taxa being differently abundant, which is in accordance with the overall clustering analyses shown above.

Functional analyses of GM confirmed the differences observed between clusters based on taxonomic composition. C2 had a higher BC to HC (0.182 ± 0.031) compared with C1 (0.146 ± 0.025) when based on abundances of KEGG orthologues (*p < *0.0001) (Supplementary Figure S4a). A higher BC to HC in C2 compared to C1 was also observed based on the abundances of CAZymes (0.234 ± 0.056 vs. 0.171 ± 0.032, *p < *0.0001) (Supplementary Figure S4b). Overall, the functional potential in samples derived from C2 was characterized by many abundance differences compared with HC, where 46.9% of all the KEGG orthologues, 58.8% of all the KEGG modules and 38.0% of all the CAZymes were higher abundant in C2 (*p *< 0.05), whereas 4.7%, 0.7%, and 9.1%, respectively, were more abundant in HC (*p < *0.05) (Supplementary Figure S4c). In contrast, there were no differences in the relative abundance of any functions between C1 and HC, further supporting the similarity between samples of this cluster with HC also on the functional level.

### TAC had a larger impact on GM composition than CSA

**Figure 3.**
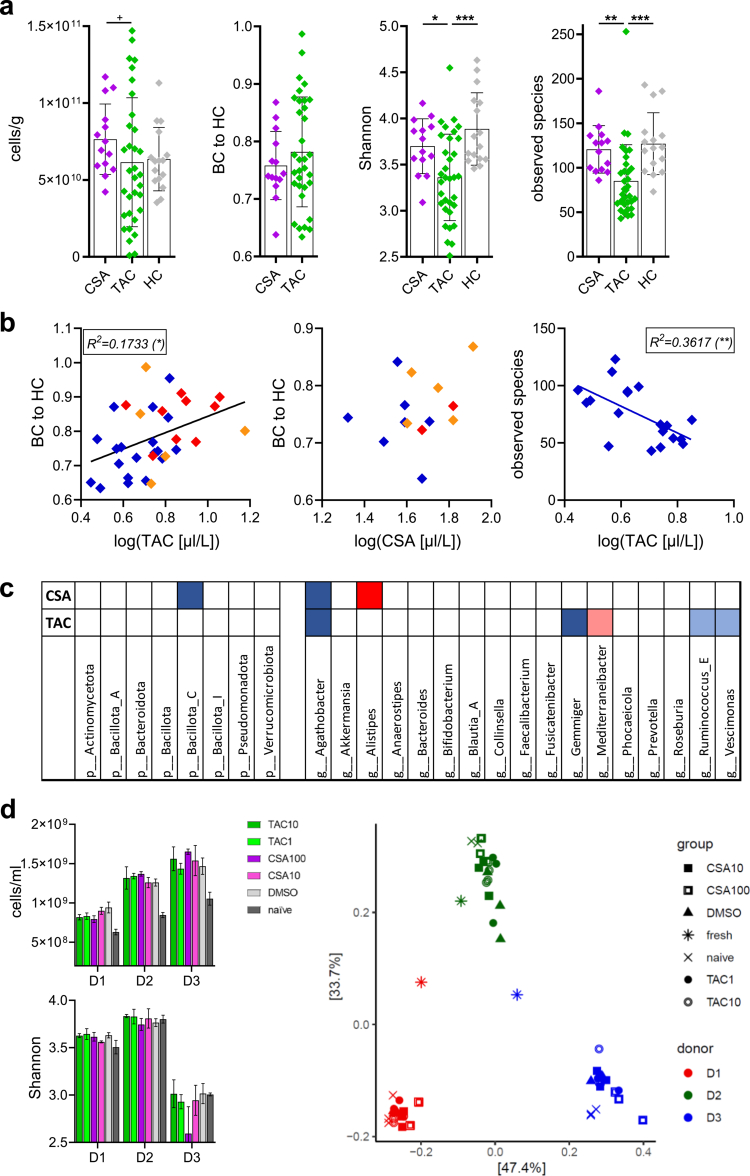
Comparison of samples based on the primary immunosuppressant administered. The distribution of the bacterial cell count, Bray–Curtis dissimilarities (BC) of each sample to the mean BC in HC (representing the degree of dysbiosis; BC to HC), the Shannon index and the number of observed species per immunosuppressant group and HC is depicted in panel a. In panel b, the association of BC to HC and tacrolimus (TAC) levels (left) or cyclosporine A (CSA) levels (center) are visualized. The right panel shows the association between TAC levels and the number of observed species in samples from liver transplant recipients (LTR). Associations are based on linear regression analyses. The table in panel c gives information about differences of the most abundant phyla (left) and genera (right) for the TAC and CSA group compared with HC (for color scheme see Figure 2). The bacterial cell counts and the Shannon index of each condition and each donor derived from *in vitro* incubations of the fecal samples are depicted in Panel d on the left side (mean ± SD, *n* = 3). The right panel depicts the bacterial composition of each sample on a metric multidimensional scaling plot based on 16S rRNA gene sequencing data. The numbers behind immunosuppressant labels indicate the concentration used [mg L^−1^]. ^+^ *p* < 0.1, *p < 0.05, **p < 0.01, ***p < 0.001, ****p < 0.0001.

Based on existing literature, we hypothesized that the type of immunosuppressant had a distinct effect on the GM composition. The results were therefore stratified based on the primary immunosuppressant, namely, TAC or CSA. There was a non-significant trend towards a lower total bacterial cell count in TAC (6.15 × 10^10^ ± 4.19 × 10^10^ cells/g) compared with CSA (7.65 × 10^10^ ± 2.29 × 10^10^ cells/g, *p = *0.0771) (Figure 3a). No difference in BC to HC was observed between the two groups. Patients receiving TAC as the primary immunosuppressant had a lower Shannon index (3.36 ± 0.47) compared with both HC (3.89 ± 0.39, *p = *0.0003) and with patients receiving CSA (3.70 ± 0.30, *p = *0.0299). Similarly, the number of observed species was lower in the TAC group (85.2 ± 40.7) compared with HC (127 ± 34.8, *p = *0.0002) and the CSA group (121 ± 26.7, *p = *0.0015). No difference in diversity between patients receiving CSA and HC were found.

We observed a positive correlation between the TAC concentration in blood and the BC to HC across all patients (*p = *0.0160) (Figure 3b, left panel); this observation lost significance when the organ was included as a random effect. Increased TAC levels were also associated with lower diversity in LTR as observed by a negative correlation between TAC blood levels and the number of observed species (*p = *0.0064) (Figure 3b, right panel). This relation was specific for LTR and was not observed when samples from HTR and KTR recipients receiving TAC were included, even when the organ was included as a random effect. The CSA levels did not correlate with any of the above analyzed variables.

Investigating GM composition showed a decreased relative abundance of *Agathobacter* (*lfdr = *0.0237) and *Gemmiger* (*lfdr = *0.0237) in patients receiving TAC as the primary immunosuppressant compared with HC (Figure 3c). Furthermore, there was a non-significant trend towards lower relative abundances of *Ruminococcus_E* (*lfdr = *0.0585) and *Vescimonas* (*lfdr = *0.0585) and a higher relative abundance of *Mediterraneibacter* (*lfdr = *0.0968). For the CSA group, a decreased abundance of bacteria belonging to the phylum *Bacillota_C* compared with HC was found (*lfdr = *0.0254). On the genus level, there was a higher relative abundance of *Alistipes* (*lfdr = *0.0166) and a lower relative abundance of *Agathobacter* (*lfdr = *0.0014) in the CSA group compared with HC (Figure 3c).

Next, we investigated whether the observed effects of immunosuppressants on the GM composition might be caused by a direct effect of the respective drugs. Incubation of fresh fecal samples with TAC or CSA did not result in differences in the total bacterial cell count, Shannon index or the number of observed species compared with the 1% DMSO control (Figure 3d). Both the immunosuppressant treated samples as well as the 1% DMSO control samples had a higher bacterial cell count compared with the naïve samples (all: *p < *0.0001), which indicated an effect of the solvent on bacterial growth. GM composition of the cultures showed clear donor-specific signatures that were in line with respective original samples (“fresh”, Figure 3d). Exposure to low concentrations of TAC and CSA, respectively, did not result in alterations of GM composition compared with 1% DMSO based on PERMANOVA analysis. Furthermore, no differences in the relative abundance of any taxon (mean relative abundance > 0.1%) were found between those groups compared with 1% DMSO. High concentrations of TAC and CSA, respectively, resulted in altered GM composition compared with 1% DMSO based on PERMANOVA analysis (TAC: *p = *0.0220, CSA: *p = *0.0010); for TAC, no taxa were found to be different, whereas for CSA, the relative abundance of three genera changed (*lfdr < *0.05), none of which were altered in the CSA group *in vivo*.

### C2 showed increased SIgA concentration and targeting

**Figure 4.**
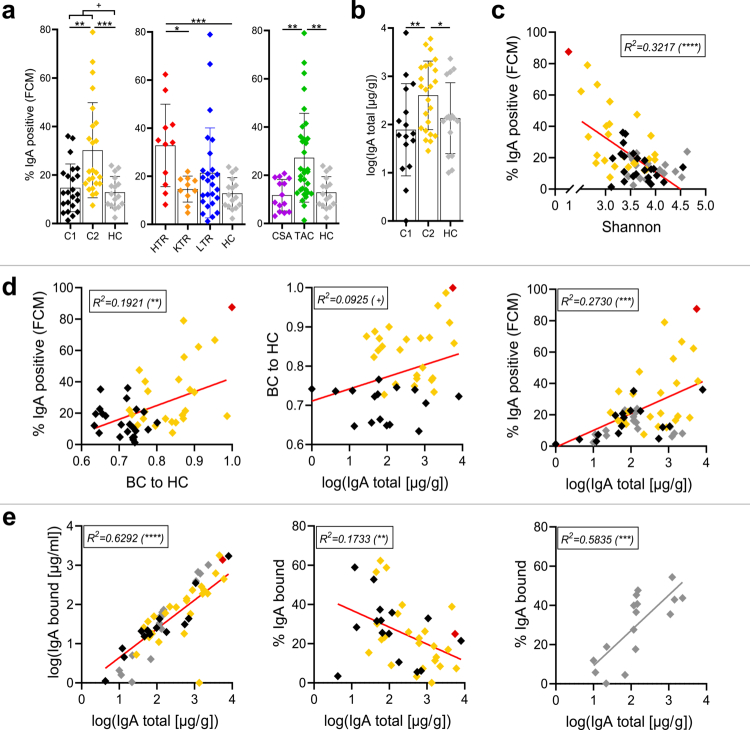
The proportion of SIgA coated bacteria (panel a) is compared between clusters (left), organ (center) and primary immunosuppressant (right). In panel b the total fecal SIgA concentration in clusters and healthy controls (HC) is shown. Panel c depicts the association of the proportion of SIgA coated bacteria and the Shannon index, whereas in panel d, the associations between the proportion of SIgA coated bacteria, total SIgA concentration and Bray‒Curtis dissimilarities (BC) of a sample to mean BC in HC (BC to HC) are compared across all samples. Panel e gives the association between the total SIgA concentration as well as the concentration (left) and percentage (center, right) of bound SIgA, respectively. Associations in panels c–e are based on linear regression analyses. The colors indicate the allocation to the respective cluster (black = C1, gold = C2, gray = HC). The sample L07 (excluded from analysis) is indicated in each graph as a red diamond. HTR = heart transplant recipients, KTR = kidney transplant recipients, LTR = liver transplant recipients.+*p* < 0.1, *p < 0.05, **p < 0.01, ***p < 0.001, ****p < 0.0001.

The specific focus of this study was to investigate whether immunosuppression affects the fecal SIgA concentration and its GM targeting. The percentage of SIgA coated bacteria ranged from 1.3% to 78.9% across all samples and, overall, patients showed a non-significant trend towards higher coating of GM by SIgA (22.6 ± 17.3%) compared with HC (12.9 ± 6.5%, *p = *0.0546) (Figure 4a, left panel). On the cluster level, C2 had a higher proportion of SIgA coated GM (30.2 ± 19.6%) compared with both C1 (14.7 ± 9.8%, *p = *0.0011) and HC (*p = *0.0002) (Figure 4a, left panel). That was accompanied by a higher total fecal SIgA concentration (1139 ± 1569 µg/g) compared with HC (445 ± 685 µg/g, *p = *0.0486) and C1 (685 ± 1969 µg/g, *p = *0.0074) (Figure 4b). On the organ level, HTR had a higher proportion of SIgA coated GM (32.8 ± 17.1%) compared with both HC (*p = *0.0006) and KTR (14.6 ± 5.4%, *p = *0.0192) (Figure 4a, center panel). KTR and LTR did not differ in their proportion of SIgA coated GM compared with HC.

Analyses based on the primary immunosuppressants revealed a higher percentage of GM coated by SIgA in patients receiving TAC (27.2 ± 18.4%) compared with patients receiving CSA (11.7 ± 6.5%, *p = *0.0019) as well as with HC (*p = *0.0036) (Figure 4a, right panel); no difference in the relative SIgA coating was found for the CSA group when compared with HC. For the sample dominated by Klebsiella (L07), the majority of bacteria (87.7%) were coated by SIgA (Supplementary Figure S2c).

A lower Shannon index was associated with a higher proportion of SIgA coated GM in patients (*p < *0.0001) but not in HC (Figure 4c). Dysbiosis (BC to HC) was positively associated with the proportion of SIgA coated GM (*p = *0.0021) and the total fecal SIgA concentration (*p = *0.0564) in patients, albeit the latter only as non-significant trend (Figure 4d). Moreover, an increase in the total fecal SIgA concentration was positively associated with an increase in the proportion of SIgA coated GM in patients (*p = *0.0005) (Figure 4d, right panel). Furthermore, there was a strong relation between the total fecal SIgA concentration and the concentration of bound SIgA (*p < *0.0001) (Figure 4e, left panel), indicating that increased SIgA secretion into the gut is indeed associated with increased targeting of the GM. However, the total fecal SIgA concentration was negatively associated with the proportion of bound SIgA in patients (*p = *0.0084), suggesting that, despite an increased SIgA binding with higher levels of total SIgA, a relative larger fraction remained not bound to bacteria (Figure 4e, center panel); this was not found in HC samples, where an increased total fecal SIgA concentration was connected with higher percentages of bound SIgA (*p = *0.0006) (Figure 4e, right panel).

### Identification of specific taxa targeted by SIgA

We characterized the GM community within the flow cytometrically sorted SIgA positive and negative fractions and compared the results with the respective total GM composition of a sample to investigate the SIgA target spectra. For the two clusters and HC we observed a separation of the whole community from both the SIgA positive fraction and the SIgA negative fraction based on PERMANOVA analyses (*p < *0.05), demonstrating specific targeting of GM by SIgA (Supplementary Figure S5). On the organ level, LTR showed, similar as HC, a separation of the total community from both fractions based on PERMANOVA analyses (*p < *0.0001). For KTR, the fraction targeted by SIgA showed a non-significant trend towards a different composition compared with total GM (*p = *0.0704) (no difference was observed with the negative fraction), whereas no overall differences between total GM composition and any fraction were seen in HTR due to highly subject-specific patterns of communities in those patients.

**Figure 5.**
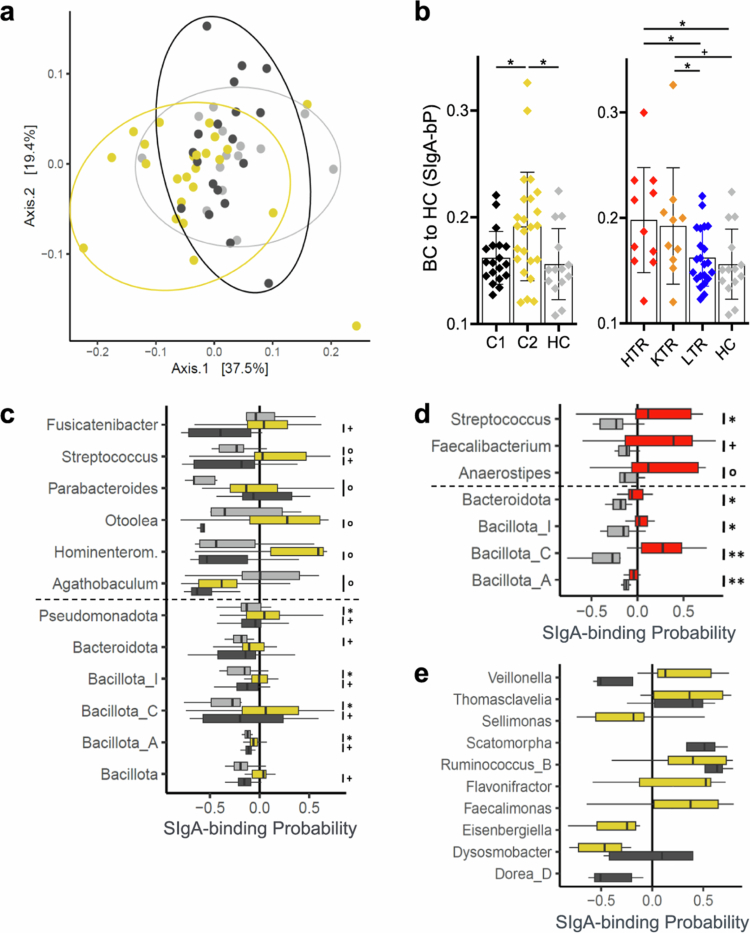
Comparison of SIgA-binding probabilities (SIgA-bP) of taxa between groups. Panel a depicts the metric multidimensional scaling analysis of all samples based on Bray‒Curtis dissimilarities (BC) of the SIgA-bP of taxa with >80% prevalence across all samples. Panel b shows the BC in each sample to the average BC in healthy control (HC) based on SIgA-bP of taxa with >80% prevalence across all samples between clusters (left) and organ (right). Taxa with different SIgA-bP between clusters and HC and between heart transplant recipients (HTR) and HC are shown in panels **c** and **d**, respectively. The dotted line (panels **c** and **d**) separates phyla from genera. Panel **e** depicts the SIgA-bP of taxa that were strongly targeted in clusters but were not abundant in HC. Colors indicate group allocation (dark gray = C1, gold = C2, red = HTR, light gray = HC). Significances: Panel b: ^+^ *p* < 0.1, *p < 0.05, **p < 0.01, ***p < 0.001, ****p < 0.0001. Panels c–e: ^o^ *p* < 0.01, ^+^ *lfdr* < 0.1, *lfdr < 0.05, **lfdr < 0.01.

To identify individual taxa that showed a shift in SIgA-targeting, we calculated the SIgA-binding Probability (SIgA-bP) as described in Jackson and colleagues.^28^ C2 showed a larger BC to HC based on SIgA-bP (0.191 ± 0.051) compared with both C1 (0.162 ± 0.025, *p = *0.0265) and HC (0.156 ± 0.033, *p = *0.0250) (Figure 5b). On the organ level, HTR had a higher BC to HC (0.198 ± 0.050) compared with LTR (0.162 ± 0.027, *p = *0.0124) and HC (*p = *0.0192). Similarly, KTR had a higher BC to HC (0.192 ± 0.055) compared with LTR (*p = *0.0469) and HC (*p = *0.0555), albeit the difference from the latter trended only and did not reach statistical significance (Figure 5b).

Next, we compared the SIgA-bP of individual taxa under the prerequisite that a respective taxon was present in at least five samples per group analyzed. The analysis of the SIgA-targeting patterns was focused on the cluster level, as previous analyses revealed that this level provided clear separation between patients and comprised high sample numbers per group. On the phylum level, C2 showed higher SIgA-bP of bacteria belonging to *Bacillota_A* (*lfdr = 0.0379*), *Bacillota_C* (*lfdr = *0.0390), *Bacillota_I* (*lfdr = *0.0379), and *Pseudomonadota* (*lfdr = *0.0390) compared with HC (Figure 5c). Furthermore, C2 showed a non-significant trend towards higher SIgA-bP across the majority of phyla compared with C1 (all: *lfdr = *0.0778) (Figure 5c). On the genus level, there were non-significant trends toward increased SIgA-bP of *Fusicatenibacter* (*lfdr = *0.0539) and *Streptococcus* (*lfdr = *0.0539) in C2 compared with C1; the latter was also more targeted in C2 when compared with HC (*p = *0.0065). Additionally, *Hominenteromicrobium* (*p = *0.0045) and *Otoolea* (*p = *0.0091) were more targeted in C2 compared with C1. Bacteria belonging to *Agathobaculum* (*p = *0.0007) and *Parabacteroides* (*p = *0.0066) were less and more targeted in C1 compared with HC, respectively (Figure 5c).

For providing comprehensive analyses, we also investigated differences in SIgA-bP on the immunosuppressant and organ levels. On the immunosuppressant level, *Bacillota_A* was more targeted by SIgA in TAC compared with HC (*p = *0.0020, Supplementary Table S2). On the genus level, *Agathobaculum* was less targeted by SIgA (*p = *0.0023), and *Parabacteroides* trended to be more targeted by SIgA in patients receiving TAC compared with HC (*lfdr = *0.0598). No differences in SIgA-bP were found in CSA compared with HC or TAC (Supplementary Table S2). On the organ level, HTR showed the most unique SIgA-targeting patterns, which included taxa that were not identified on any other level. On the phylum level there were strong increases in SIgA-bP of the taxa *Bacillota_A* (*lfdr = *0.0085), *Bacillota_C* (*lfdr = *0.0091), *Bacillota_I* (*lfdr = *0.0180), and *Bacteroidota* (*lfdr = *0.0180) in HTR compared with HC (Figure 5d). On the genus level, *Faecalibacterium* (*lfdr = *0.0530) and *Streptococcus* (*lfdr = *0.0267) were more targeted in the HTR compared with HC, although the former only as a non-significant trend. Additionally, *Anaerostipes* showed an increased targeting in HTR compared with HC (*p = *0.0095) (Figure 5d). No difference in SIgA-bP was found in LTR and KTR, except for a higher SIgA-bP of *Agathobaculum* in LTR (*p = *0.0057) (Supplementary Table S2).

Finally, we investigated taxa that were specifically associated with patients' GM and therefore contributed to the observed dysbiosis in SOT recipients. We focused on taxa that were present only in patients or a specific patient cluster and largely devoid in HC and selected those that were overall strongly or hardly targeted by SIgA as defined by a SIgA-bP ± 0.25. The taxa *Ruminococcus_B* and *Thomasclavelia* were present in the majority of patient samples (*n* = 32 and *n* = 30, respectively) and were mostly absent in HC. Both taxa were highly targeted by SIgA, with an average SIgA-bP of 0.42 ± 0.38 and 0.32 ± 0.32, respectively (Figure 5e). Bacteria belonging to the taxa *Dysosmobacter* and *Veillonella* were present in the majority of patients and but only showed a strong negative SIgA-bP in one cluster, namely, *Dysosmobacter* in C2 and *Veillonella* in C1 (Figure 5e). Four taxa were predominantly present in C2, of which two showed strong positive SIgA-bP values, namely, *Faecalimonas* and *Flavonifractor*, whereas *Eisenbergiella* and *Sellimonas* had a strong negative SIgA-bP in C2. C1 was characterized by a strong positive SIgA-bP of *Scatomorpha* and a strong negative SIgA-bP of *Dorea_D*, both of which were hardly abundant in C2 (Figure 5e).

## Discussion

Life-long immunosuppression after SOT is crucial to prevent the rejection of the transplanted organ. However, it is also associated with alterations in GM composition.^8^ We confirmed persisting GM dysbiosis in SOT recipients even several years post-transplantation and showed that the extent of GM dysbiosis was associated with the overall immunosuppressant regimen, in particular with TAC levels. Additionally, we demonstrated that the total SIgA concentration and the proportion of SIgA coated bacteria are elevated in those patients, which was associated with the degree of dysbiosis and altered SIgA target spectra.

The observed GM dysbiosis in our cohort is in accordance with existing literature investigating the GM composition in human adult and pediatric SOT recipients.^29^ ^,^ ^30^ Identifying factors contributing to GM dysbiosis is crucial for the short- and long-term health of SOT recipients. In the short-term post-surgery period, GM dysbiosis is associated with several complications, including an increased risk for infection,^12^ diarrhea^29^ ^,^ ^31^ and acute rejection (as reviewed in^10^ ^,^ ^32^). Regarding long-term development, we recently demonstrated an inverse correlation between GM diversity and transaminase levels as indicator for graft (liver) health and were further able to predict hepatocellular damage of the transplant based on the GM with high accuracy.^2^ A recent large-scale analysis of 1337 metagenomes from a longitudinal follow-up cohort by Swarte et al.^11^ further highlighted the relevance of targeting GM dysbiosis in SOT recipients, as dysbiosis was strongly positively associated with long-term all-cause mortality as well as cause-specific mortality in those patients.

**Figure 6.**
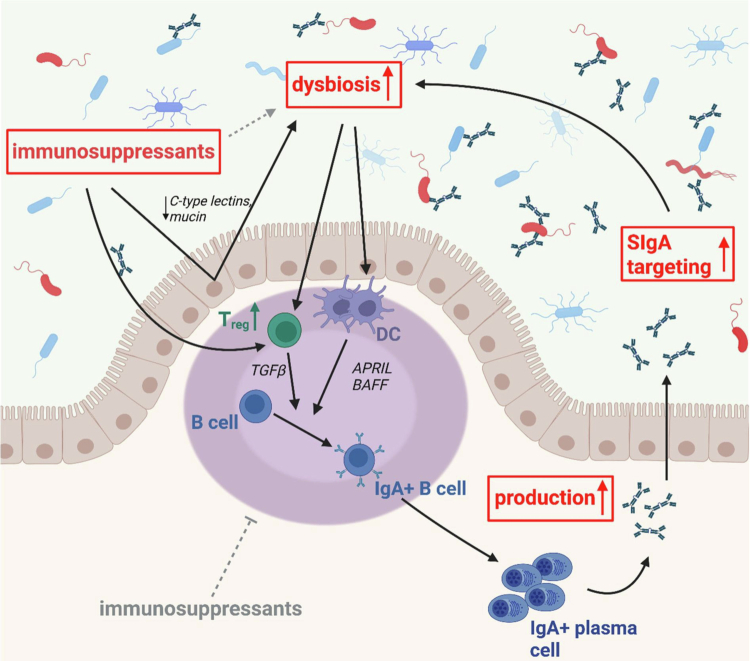
Proposed model connecting immunosuppression, gut microbiota (GM) dysbiosis and SIgA-targeting in SOT recipients; parameters in red were measured in this study. Drug-induced immunosuppression together with increased SIgA concentration and altered targeting are all contributing to dysbiosis in SOT recipients. Two potential routes explaining the observed higher SIgA production are discussed. (1): T-cell independent B cell class switch that is activated via microbial stimulation of dendritic cells (DCs) and subsequent expression of A proliferation-inducing ligand (APRIL) and B cell-activating factor (BAFF). (2): T-cell dependent B cell class switch as a result of an increased activity of regulatory T cells (*T*_reg_) promoted by both immunosuppressants and dysbiosed GM.

The initial hypothesis of this study was that immunosuppression therapy results in a reduced SIgA production and GM targeting, as it acts on T and B cell proliferation and differentiation, which are important for IgA production. However, the obtained results contradicted this hypothesis. In contrast, we observed higher fecal SIgA concentration and GM targeting in SOT recipients, which were further associated with higher immunosuppressant levels as well as with increased numbers of different types of immunosuppressants. We created a hypothetical model connecting drug-induced immunosuppression, the GM composition and SIgA-targeting in order to integrate our results into a larger mechanistical framework based on the existing literature (Figure 6). First, we observed that the extent of GM dysbiosis in SOT recipients was associated with increased immunosuppressant levels, at least with TAC. We collected samples at least one year post-transplantation to exclude any direct influences on the GM from surgery and associated short-term (antibiotic) treatment thereafter and further excluded any patients with recent antibiotic intake enabling the hypothesis that GM dysbiosis in our cohort was indeed caused mainly by immunosuppressant-mediated effects. We observed a strong and blood level-dependent effect of TAC on GM dysbiosis that was not observed in patients receiving CSA as the primary immunosuppressant. Furthermore, the multidrug-regimen in HTR and KTR might have contributed to the higher GM dysbiosis in those patients compared with LTR, however, the large heterogeneity of administered drug-combinations in those patients prevented further exploration of individual drug-mediated effects aside from TAC and CSA. The association between drug-induced immunosuppression and GM dysbiosis was already observed in earlier studies based on both animal models and humans.^33^ ^-^ ^35^ Still, as most studies were observational, the question of the underlying mechanism by which immunosuppressants affect the GM composition remained open. We observed small direct effects of TAC and CSA on the GM composition at high concentrations. However, it is unlikely that the GM areexposed to such high concentrations *in vivo* as most is absorbed at proximal sites in the GI tract. Maier and colleagues^36^ screened non-antibiotic drugs regarding their impact on selected GM strains in pure culture and saw a small effect of CSA on five of the 40 total strains tested at a concentration of 24 mg L^−1^; the authors did not investigate TAC. Another route for drugs alter the GM composition might be via the immune system, which has been the focus of a very few studies. For instance, Tourret and colleagues^37^ found that altered GM composition was associated with reduced expression of C-type lectins and IL-22 after 14-d upon treatment with TAC or prednisolone, among others. Another study in mice found that treatment with the glucocorticoid dexamethasone resulted in decreased colonic Muc2 gene expression and a shift in GM composition.^38^ In general, disentangling the direct effects of immunosuppressants on the GM from effects mediated via alterations of the immune system is a difficult task, and the contribution of each route to dysbiosis remains largely elusive. This problem was also discussed in a systematic review from Manes and colleagues^34^, who compared existing studies to investigate the bidirectional interaction between immunosuppressants and the GM. The authors highlighted that the results were largely based on observational studies and that the comparison of the results was heavily confounded by the heterogeneity of the administered drug(-combinations), the inclusion and exclusion criteria, the overall study design as well as GM modulating effect of the underlying disease itself. In accordance, Gabarre and colleagues^4^ highlighted the complexity of targets by which immunosuppressants can interfere with GM-immune system interaction thereby making it difficult to identify whether a change in GM or a specific bacterium is a direct consequence of such an interference.

GM dysbiosis in patients was positively associated with increased and altered targeting of GM by SIgA in our cohort and especially pronounced in patients allocated to C2. Patients in this cluster were characterized by an overall increase in the fecal SIgA concentration and proportion of SIgA coated bacteria as well as specific alterations in SIgA-targeting patterns. The association between higher SIgA levels and GM dysbiosis has already been observed in other disease cohorts, e.g. inflammatory bowel diseases (IBD),^39^ ^,^ ^40^ psoriatic arthritis^41^ and schizophrenia.^42^ However, the question of how increased SIgA-targeting of the GM affects dysbiosis was not addressed in these studies. SIgA-targeting is an important host-mediated mechanism for pathogen exclusion and the promotion of a balanced and healthy GM community.^43^ ^,^ ^44^ Consequently, one can hypothesize that the host immune system increases specific SIgA production to resolve drug-related GM dysbiosis. Based on this hypothesis, we expected to observe higher SIgA-targeting of GM members that showed increased relative abundances in patients compared with HC. However, we found increased SIgA-targeting of several commensals that were not differently abundant between patients compared with HC. This observation does not substantiate the hypothesis that altered SIgA-targeting of the GM in patients is a specific response to conquer GM dysbiosis. Rather, our data indicate that increased SIgA production and target spectra are the consequence of a dysfunctional immune system resulting in the production of more low-affinity and polyreactive SIgA, a feature that is associated with targeting of commensals,^45^ and hence, contribute to GM dysbiosis in SOT recipients (Figure 6).

We found higher SIgA production despite an expected immunosuppressant-mediated suppression of T and B cell activation and proliferation.^46^ ^-^ ^49^ While T cell dependent IgA class switching is considered the major route for the generation of IgA+ B cells, T cell independent routes exist as well and potentially contributed to the increased SIgA concentration observed in this study. T cell independent IgA class switching is mediated by dendritic cells (DCs) that sense microbial toll-like receptor ligands.^45^ DCs then express intestinal nitric oxide synthase (iNOS) that subsequently stimulates the production and release of two tumor necrosis factor (TNF) family members, namely A proliferation-inducing ligand (APRIL) and B cell-activating factor (BAFF).^45^ ^,^ ^50^ Binding with their respective receptors on B cells triggers IgA class switch (Figure 6).^50^ Another study showed that also intestinal epithelial cells (IECs) secrete APRIL after sensing microbial products and additionally increase APRIL production by stimulating DCs.^51^ While T cell dependent responses result in the production of highly specific SIgA, T cell independent responses are associated with the production of low-affinity, polyreactive SIgA.^22^ ^,^ ^52^ ^,^ ^53^ An increase in polyreactive SIgA might also explain the increased SIgA-targeting of commensals in our cohort as mentioned above further supporting a prominent role of microbiota-mediated, T cell independent generation of IgA+ plasma cells. Determining the exact target spectra of IgA would further contribute to our understanding of the observed results. For example, experiments incubating different, synthetic communities resembling those of patients with isolated free SIgA from patients and HC, respectively, would allow us to investigate altered SIgA-targeting in more detail but exceeded the scope of this study.

Direct effects of immunosuppressants on B cell CSR might also have contributed to our findings. Observations from the literature link immunosuppressants and GM dysbiosis to an increased expression of regulatory T cell (*T*_reg_) that were found to promote IgA class switch and subsequent IgA production via transforming growth factor β (TGFβ) (Figure 6).^54^ ^-^ ^56^ Several immunosuppressants were shown to directly increase *T*_reg_ expression. TAC was associated with increased *T*_reg_ numbers and activity both *in vitro* ^57^ as well as in KTR comparing *T*_reg_ numbers before and four months after transplantation.^58^ Similar results were found for prednisolone.^59^ ^,^ ^60^ In contrast, CSA was found to inhibit *T*_reg_ function both *in vitro* ^61^ and *in vivo,* ^62^ which supports a *T*_reg_ mediated increase in SIgA production in our cohort, as we did not see increased SIgA concentrations in patients receiving CSA as the only immunosuppressant. Increased *T*_reg_ numbers were also found as a consequence of an altered GM after TAC administration that was clearly separated from a direct drug-mediated effect.^63^ However, to verify whether *T*_reg_ indeed contributed to the observed increased SIgA production, investigations of blood or tissue samples would have been required.

Taken together, our model suggests that long-term intake of immunosuppressants is associated with GM dysbiosis and that increased and altered SIgA-targeting of the GM in pediatric SOT recipients. Based on these findings and observations from the literature, we hypothesize that continuous intake of immunosuppressants is causing GM dysbiosis and that both GM dysbiosis and immunosuppressants stimulate SIgA production. T cell independent, DC-mediated IgA class switch along with stimulation of the IgA class switch by immunosuppressants via *T*_reg_ are potentially contributing. However, the exact underlying immunological mechanisms remain speculative. We further postulate that the increased and altered SIgA-targeting of the GM enhances GM dysbiosis, creating a positive feedback loop where SIgA-mediated dysbiosis is fueling increased SIgA production along with alterations in their target spectra (Figure 6).

This study has some limitations because of its design as a mono-centric study limiting the total number of patients and resulting in a lower sample size. Nevertheless, by restricting the study to one site, we minimized confounding factors such as the regional variability of the GM as well as differences in surgical and post-surgery management, thus allowing a direct comparison of the different groups and providing the first major insights into the role of SIgA at the interface between the GM and the host immune system in SOT recipients. We observed a high heterogeneity of immunosuppressant regimens, especially in KTR and HTR, which might have masked some effects from individual primary immunosuppressants. Higher sample numbers would allow a more in-depth analysis of the contribution of each immunosuppressant to the observed effects. We collected only one sample per patient, thus performing cross-sectional analyses across organs and immunosuppressant groups. While it allowed for uncovering major signals, a longitudinal approach would provide the basis for more detailed analyses on SIgA responses in the context of GM dysbiosis development. Finally, we were not able to investigate the detailed underlying immunological mechanisms explaining our observations, as this would have required additional analyses of tissue and/or blood samples that were not available.

In summary, while immunosuppressants are administered as a life-long therapy to prevent graft rejection, they are also associated with GM dysbiosis that remains stable even years after transplantation, thereby increasing the risk for (long-term) complications. Our study highlights the crucial role of SIgA at the interface between the host immune system and the GM in SOT recipients and suggests a contributing role of SIgA for the observed GM dysbiosis in those patients. These results give rise to SIgA as a novel factor contributing to GM dysbiosis in SOT recipients.

## Supplementary Material

Supplementary Figures.pdf

Supplementary material Supplementary Table S2_Difference in SIgAbP.xlsx

Supplementary material Supplementary Table S1_Difference in abundance.xlsx

## Data Availability

All sequences are publicly available at the European Nucleotide Archive (PRJEB106142).

## Abbreviations

APRIL: A proliferation-inducing agent
BAFF: B cell-activating factor
BC: Bray–Curtis dissimilarity
CSA: cyclosporine A
DC: dendritic cell
ELISA: enzyme-linked immunosorbent assay
GM: gut microbiota
HC: healthy control
HTR: heart transplant recipients
IEC: intestinal epithelial cell
KTR: kidney transplant recipients
*lfdr*: local false discovery rate
LTR: liver transplant recipients
MMF: mycophenolate mofetil
SIgA: secretory immunoglobulin A
SIgA-bP: SIgA-binding probability
SOT: solid organ transplantation
TAC: tacrolimus
TGFβ: transforming growth factor β
TNF: tumor necrosis factor
*T*_reg_: regulatory T cells
