# Supplementary material for: Altered SIgA-targeting of gut microbiota is associated with long-term dysbiosis in pediatric solid organ transplant recipients
Source: Gut Microbes. 2026 May 24;18(1):2675078. doi: 10.1080/19490976.2026.2675078 (PMC13203069; doi:10.1080/19490976.2026.2675078)
Supplement: Supplementary Figures.pdf [file KGMI_A_2675078_SM8398.pdf]

Supplementary Figures

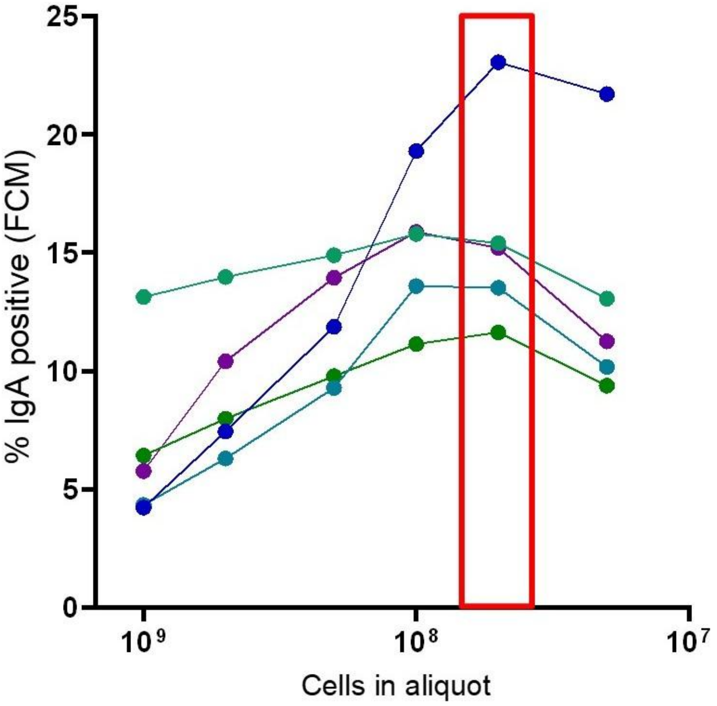

Supplementary Figure S1. Comparison of different bacterial cell counts used during the establishment of the protocol for the quantification of SIgA-coated bacteria. Each color represents one fecal sample. 50 million bacteria were found as an optimal number to determine SIgA-coated bacteria (red box).

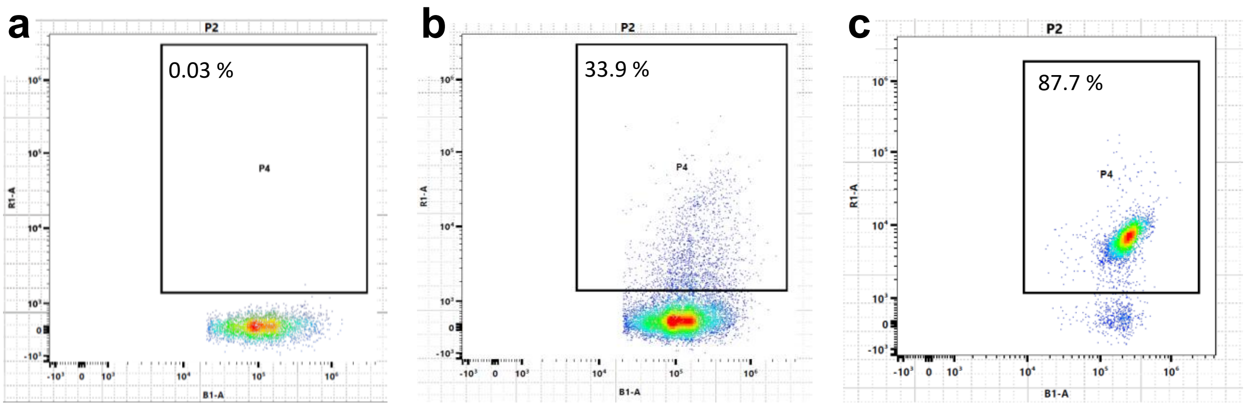

Supplementary Figure S2. Representative images of flow cytometric analyses of a sample before (panel **a**) and after (panel **b**) incubation with the anti-IgA antibody. Gate P4 included all

bacteria that were bound by the anti-IgA antibody. Percentages indicated the relative proportion of bacteria in P4. For sample L07, most bacteria (87.7 %) were strongly SIgA positive (panel **c**).

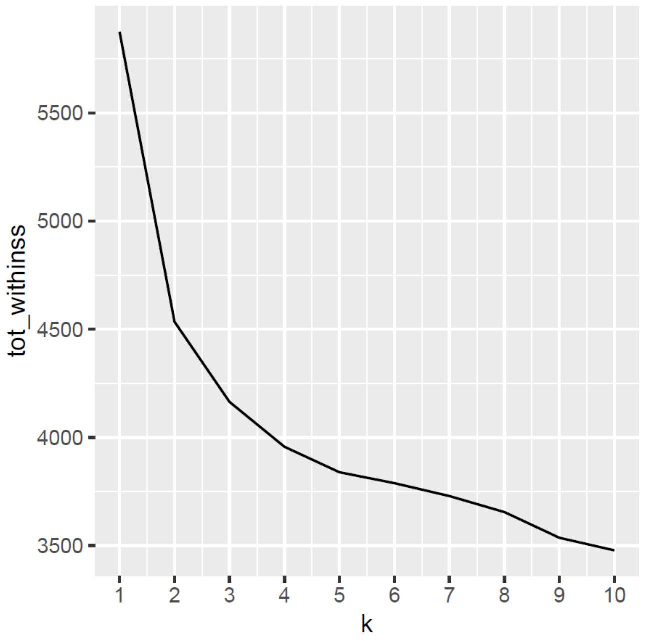

Supplementary Figure S3. Elbow plot from k-means based clustering algorithm to identify the optimal number of clusters using metagenomics data on species level and after the exclusion of sample L07. According to this analysis a k of 2 is optimal to stratify samples.

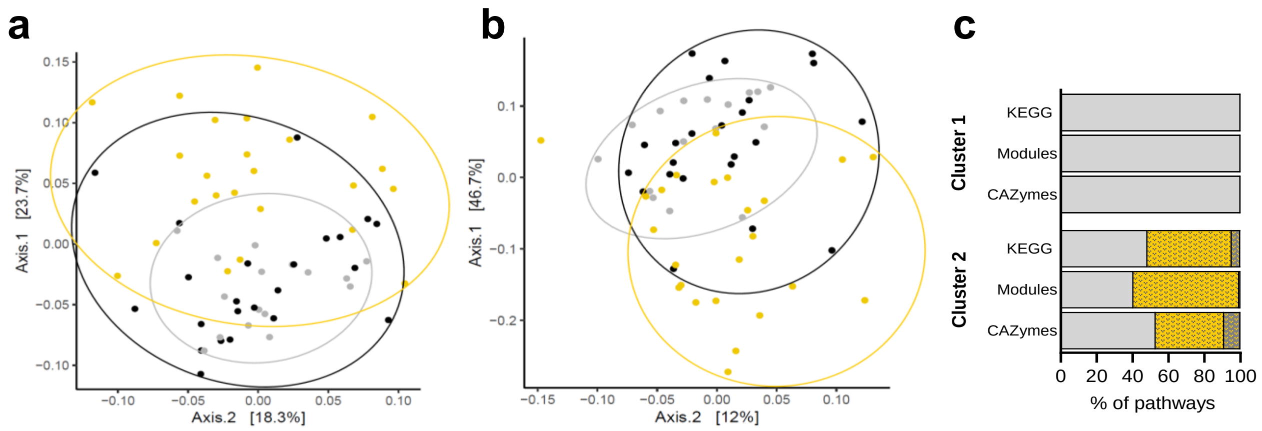

Supplementary Figure S4. Functional analysis of bacterial communities based on metagenomics data. Metric multidimensional scaling analysis of all samples based on Bray Curtis dissimilarities of the abundances of KEGG orthologues (panel **a**) and CAZymes (panel **b**). Samples are colored based on the allocation to the identified clusters from the taxonomy-based analysis shown in Figure **1a** and the respective cluster distribution was further visualized by circles. Panel **c** indicates the percentage of pathways with a similar (light grey), higher (gold) or lower (dark grey) abundance in patient clusters compared with healthy controls (HC).

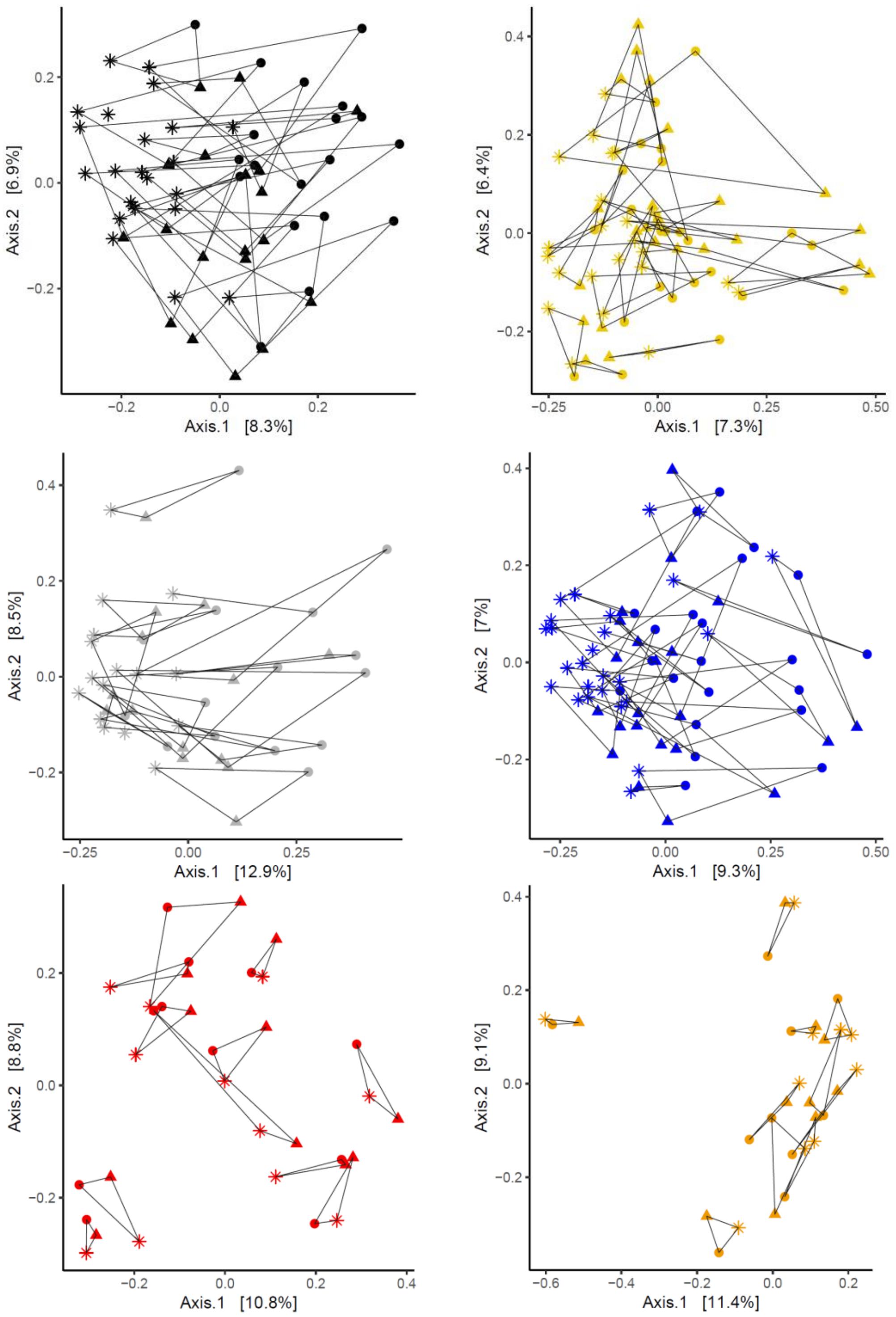

Supplementary Figure S5. Metric multidimensional scaling analysis based on Bray Curtis dissimilarities of 16S rRNA gene sequencing data comparing bacterial compositions of the unsorted samples (star), the positive fractions (dot) and the negative fractions (triangle) per cluster (upper panels) and per transplanted organ (lower panels). Lines are connecting sample of each subject. Samples are color coded according to groupings shown in Figure **1**.
